# Effect of Total Hip Arthroplasty with Ceramic Acetabular Component on Clinical, Radiographic and Functional Parameters in Older Patients with Hip Osteoarthritis: Two-Year Follow-Up

**DOI:** 10.3390/jcm12020670

**Published:** 2023-01-14

**Authors:** Alexandre Penna Torini, Carlos Eduardo Barsotti, Rodrigo Mantelatto Andrade, Luiz Henrique da Silva Nali, Ana Paula Ribeiro

**Affiliations:** 1 Biomechanics and Musculoskeletal Rehabilitation Laboratory, Health Science Post-Graduate Department, School of Medicine, University Santo Amaro, São Paulo 04829-300, Brazil; 2 Spine and Hip Group, Hospital Beneficência Portuguesa, São Paulo 01323-001, Brazil; 3 Medicine and Physical Therapy Department, School of Medicine, University of Sao Paulo, São Paulo 05360-160, Brazil

**Keywords:** arthroplasty, hip, osteoarthritis, pain, instability, functionality

## Abstract

Background: Total hip arthroplasty (THA) is a widely used surgical procedure to reduce pain and improve function and quality of life in patients with hip disorders. The most common condition that leads to THA is osteoarthritis, with most surgeries being performed to treat severe osteoarthritis with pain and functional limitations. Despite the evident success of THA, the search for its improvement and better results, especially in the long term, continues, especially in older patients, for which there is still little scientific evidence. Objective: To evaluate the clinical, radiological, and functional aspects preoperatively and two years after THA with a ceramic acetabular component device in older patients with hip osteoarthritis. Methods: A retrospective cohort study was conducted to evaluate 65 older individuals who underwent THA of the hip with an acetabular component (MD^®^ ceramic head with a ceramic acetabular insert) associated with the MD6^®^ Phenom^®^ femoral rod type, in Hospital of the Luz, São Paulo/SP, between 2018 and 2019. Anthropometric and clinical information about the operative procedure and two years follow-up were collected from the patients’ medical records. For the clinical-functional evaluation, the Harris Hip Score (HHS) questionnaire and hip movement goniometry were applied. For the radiographic parameters, the following were evaluated: the positioning of the acetabular component, the Zone of DeLee and the offset of the femoroacetabular component. Results: There was a higher prevalence of performing THA in males (53.8%). Preoperative and two-year postoperative radiographic parameters of surgical treatment for THA showed maintenance of the acetabular (*p* = 0.083) and femoral (*p* = 0.102) positioning angles and increased functionality (*p* < 0.001) and joint mobility of the hip (*p* = 0.001) with reduced pain after two years of THA. Complications related to dislocation, loosening, infection, and inadequate positioning of the implant were low, ranging from 1.5 to 3%. Conclusion: Older people who underwent cementless THA with an ceramic acetabular component device, in a two-year follow-up, showed effectiveness in improved clinical, radiological, and functional aspects.

## 1. Introduction

Hip osteoarthritis (HOA) is a complex chronic disease characterized by degeneration of articular cartilage, subchondral bone thickening, and osteochondral proliferations [1,2], whose involvement is greater in men aged up to 45 years and in women aged over 60 years [3]. It is a process characterized by alterations in the structure and function of the hip joints, which results in major pain symptoms and functional limitations in affected patients [1,4,5]. 

Currently, OA affects approximately 250 million people worldwide, including adults and older adults, and is considered to have a major impact on public health [2,6], with a medical cost directed at the disease in several high-income countries reaching estimates of 1% to 2.5% of the gross domestic product of these countries. Surgical treatment with total hip arthroplasty (THA) represents the largest proportion of these health services and costs [7,8,9,10]. THA is certainly one of the great triumphs of orthopedic medicine [4,6,11,12] and of the health system as a whole [10,13,14]. For a long time, people with severe hip illnesses were destined to live their lives with major pain conditions. THQ drastically changed this framework. Many debilitated patients who in the past needed crutches or a wheelchair have obtained a new, promising, and exciting treatment [15,16]. This procedure allows these patients to remain independent, without burdening their families and society. Few surgical procedures have been so successful for both the patient and society as a whole. Currently, one million THAs are carried out annually around the world [16].

Despite the high prevalence of THA, conservative treatment should be tried before surgical indication, involving weight loss, systemic or local medications, physiotherapy exercises (exercises in mobility, muscle strength, balance, and gait), the use of an orthosis (cane), and guidelines for the practice of physical activity [4,13,17]. Thus, the indication of THA should be based on the failure of conservative treatment and on a justifiable clinical framework. The primary indication is debilitating pain, which worsens with physical activity and improves with rest. Decreased joint range of motion is also an important factor. This surgery is traditionally reserved for older adults due to the wear of the components; however, with technological advances, it is increasingly performed in young people [11,18,19].

The goals of THA are therefore to relieve pain and improve joint function [11,19]. THA is a joint replacement surgery, involving an acetabular and a femoral component, which must be fixed to the pelvic and femoral bones, aiming at the best possible position to ensure proper functioning of the prosthesis. The acetabular component, also known as the acetabular cup, can be single and cemented to the acetabulum or it can have two parts: an external dome fixed without cement through impaction (“pressfit”) and/or screws and an internal component (“liner” or “insert”) embedded in the external component. The femoral component is generally made up of a rod that can also be cemented or fitted without impaction cement, and a head that fits onto the rod [11,19].

Correct positioning of the acetabular component is essential for the biomechanical stability of THA, ensuring long-term survival and avoiding dislocation, which occurs when the head of the femoral component disengages from the acetabular component [19,20,21,22,23,24,25,26]. The dislocation rate in primary THAs is between 1% and 4% and may reach 10% in the case of revisions [27,28,29,30,31,32]. Of the 51,345 THA revisions performed in the United States between October 2005 and December 2006, the largest cause was dislocation (22.5%), with an average cost per patient of USD 54,553 [33]. Several authors believe that poor positioning of the acetabular component is responsible for most dislocations [11,24,30,34,35].

Currently, the positioning of the acetabular component is performed through the surgeon’s experience and conventional mechanical guides. These guides are not accurate, as they assume that the patient’s trunk and pelvis are aligned in a known orientation on the operating table, without taking into account the distinctive variations in each individual, the actual position of the pelvis on the operating table, and the various possible intraoperative movements [18,20,21,24,30,36,37,38,39]. Thus, clinical follow-up and imaging exams are important resources used in post-THA surgery to monitor the positioning of prostheses. 

Despite the advances of uncemented prosthesis, there is still a discussion in the literature regarding the use of this model in patients with osteoarthritis from rheumatologic causes, mainly hip osteoarthritis in an advanced stage that affects elderly patients. However, some studies have supported the use of cementless prostheses for functional improvements in these patients [40,41,42]. The understanding of the uncemented prosthesis with the use of tribological pair (ceramic head with ceramic acetabular), produced in Brazil, to promote low-cost care in public health hospitals, was what encouraged this study.

Image parameters are an important tool, used to identify the behavior of implanted devices, as well as joint restoration, aiming at the stability of the arthroplasty [32]. Even with the evident success of THA, the search for its improvement and better results, especially in the long term, continues, mainly in the development of new surfaces, materials with greater biocompatibility, and less aggressive surgical techniques [11,24]. This fact justifies the clinical relevance of the present study which proposes to analyze, in the short and medium term, clinical and radiological aspects of patients who underwent THA, using devices of MD^®^ national components. Thus, the aim of this study was to evaluate the clinical, radiological, and functional aspects preoperatively and two years after THA with a ceramic acetabular component device in older patients with hip osteoarthritis.

## 2. Materials and Methods

### 2.1. Study Design and Participants

This is a retrospective cohort study where 65 older patients with hip osteoarthritis, in advanced stage of the disease, who underwent a hip arthroplasty (THA) with primary cementless total hip prosthesis using ceramic acetabular component devices, this surgical procedure being performed by the same team of surgeons. These patients were evaluated between the years 2018 and 2020 at the Hospital of the Luz in the city of São Paulo/SP were evaluated and clinical, radiological, and biomechanical-functional aspects were monitored through a medical consultation at the Orthopedic Clinic. The sample size is based on the literature, which uses a smaller sample number of patients with rheumatologic causes who underwent cementless THA [40,41,42]. This study was approved by the Research Ethics Committee of University local under number: 4.091.002. All patients signed an informed consent form to authorize their participation and the assessments, in accordance with resolution 466/12 of the National Health Council. 

Inclusion criteria were: older patients with hip osteoarthritis, in an advanced stage of the disease, undergoing THA; having a primary cementless total hip prosthesis with MD^®^ ceramic acetabular component devices (ceramic head with ceramic acetabular insert and Phenom^®^ MD6^®^ femoral type design); aged between 60 and 80 years; with THA performed at the private Hospital of the Luz in the city of São Paulo/SP with the same team of surgeons; being a patient under regular follow-up at the orthopedic clinic; preserved cognitive ability; and availability to attend periodic reassessments. Exclusion criteria were: not agreeing to participate, not presenting or undergoing a follow-up X-ray, bilateral hip osteoarthritis, and having a cemented hip prosthesis after femur fracture, as a primary or secondary form. 

### 2.2. Analysis of Medical Records

**Figure 1:**
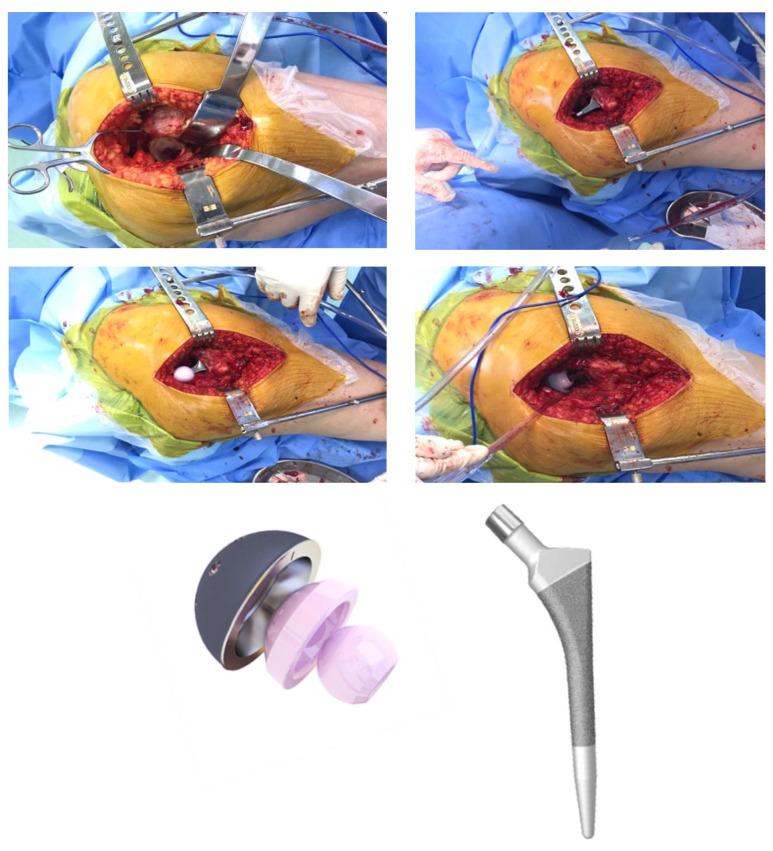
Underwent THA using the MD^®^ ceramic acetabular component devices (ceramic head with ceramic acetabular insert) associated with the MD6^®^ Phenom^®^ femoral rod type.

Retrospectively, during the period of 24 months, referring to the years 2018 and 2019, a survey was carried out from the medical records of patients who underwent THA using the MD^®^ ceramic acetabular component devices (ceramic head with ceramic acetabular insert) associated with the MD6^®^ Phenom^®^ femoral rod type (Vincula^®^ Ind. Com. Imp. Exp. Implants, DT^®^ industrial, Rio Claro, São Paulo, Brazil, https://vincula.com.br/sobre-a-vincula/?lang=en (accessed on 30 December 2022) at the Hospital da Luz, in the city of São Paulo/SP (Figure 1). After selecting the medical records, the following information was tabulated: sex, age, date and diagnosis of admission, type of THA surgical procedure performed, and type and size of the prosthesis used. In addition, in the evaluation of outpatient monitoring, clinical complications related to loosening and dislocation in the two-year postoperative period were collected.

### 2.3. Analysis of Radiographic Parameters: Positioning and Inclinations of the Acetabular Component

Variables related to component instabilities were also evaluated, such as implant fixation and positioning [32]. For these assessments, the patient was invited, by telephone, to a medical consultation, to which they were asked to take the first preoperative radiograph of the THA, followed by other radiographic images, 24 months after the surgical procedure. 

**Figure 2:**
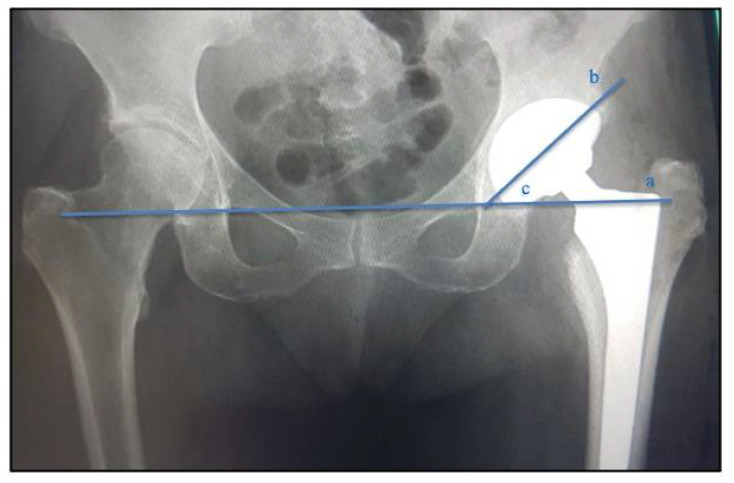
Acetabular component positioning angle measurement. (a)—line that touches the ischial tuberosities; (b)—line through the axis of the largest diameter formed by the projection of the metallic ring on the radiograph; (c)—acetabular angle.

In the radiographic image exams, preoperatively and two years after the surgical THA procedure, the positioning of the acetabular component was evaluated. To measure the acetabular position, the patient was placed in dorsal decubitus with the radius centered on the pubic symphysis, showing both hips (obturator foramen equal on both sides) and including the proximal third of the femur. To verify the positioning of the acetabular component, the angle between a line that joined the ischial tuberosities and a line that crossed the long axis of the acetabular component was measured, determined by means of the axis of the largest diameter formed by the projection of the metallic ring on the radiograph [32] (Figure 2). 

**Figure 3:**
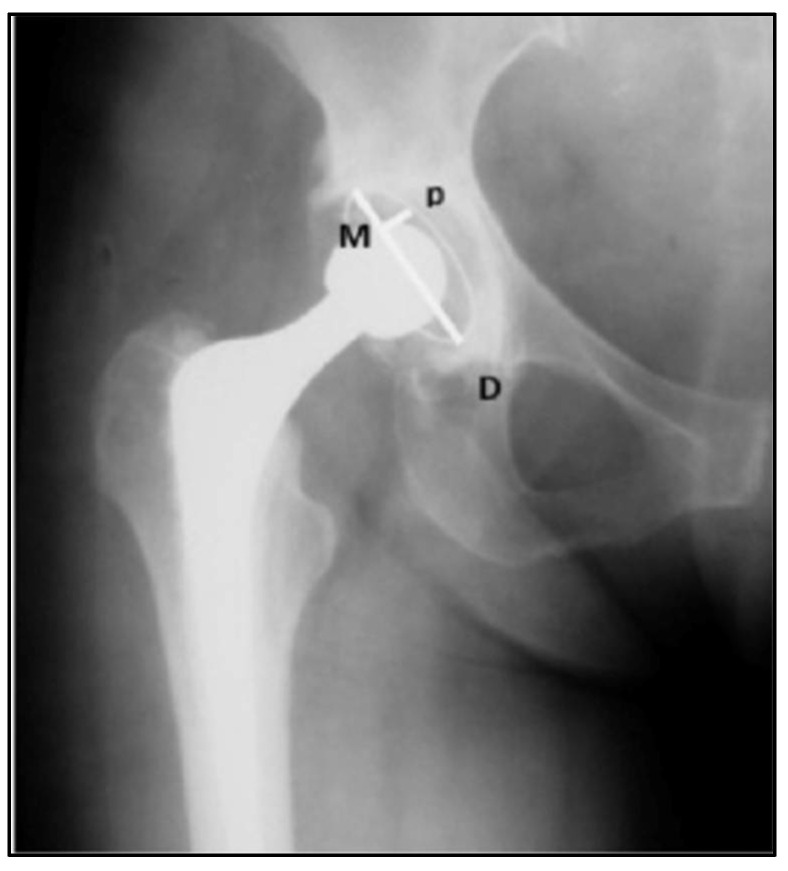
Radiographic areas of acetabular displacement, proposed by DeLee and Charnley, represented in the angle of lines M and D (1976) [43].

Another important parameter that was evaluated was the DeLee and Charnley Zone (1976) [43], characterizing the existence or not of loosening of the acetabular component, as long as it was identified in the medium-term postoperative radiographs. Thus, the DeLee and Charnley Zones are three areas described around the acetabular component, which may present osteolysis and indicate partial (when affecting only zones 1 and 2) or complete loosening (when affecting all three zones) (Figure 3).

**Figure 4:**
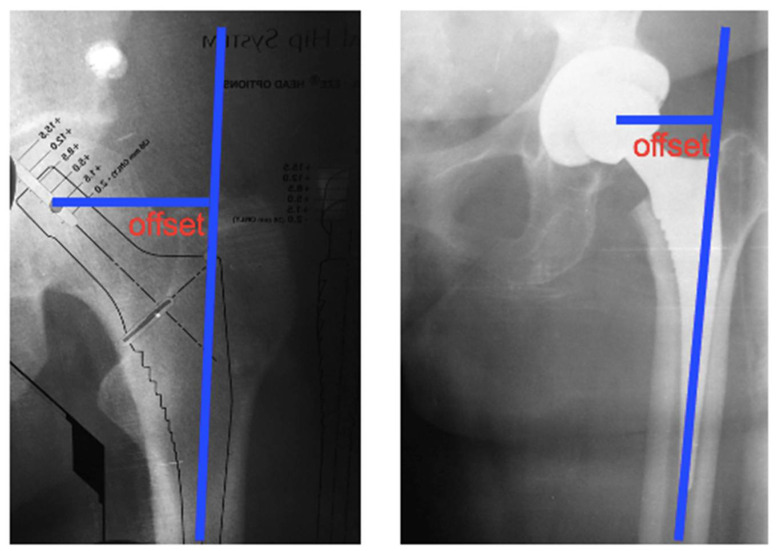
Offset of the femoral component; distance from the center of the femoral head to the axis line of the distal part of the rod.

Femur offset was also evaluated, defined as the distance from the center of rotation of the femoral head to a line drawn on the long axis of the femur. This measurement varies according to hip rotation. Next, the offset of the femoral component was measured, characterized by the horizontal deviation of the distance from the center of the femoral head to the axis line of the distal part of the rod. Improper offset restoration shortens the abductor musculature lever and results in increased joint reaction force, flaccidity, and bone contact, which can result in joint displacement (Figure 4). 

### 2.4. Functional Parameters of the Hip

For functional analysis of the hip, the Harris Hip Score (HHS) questionnaire was applied, a specific instrument for evaluating the hip, with regard to the domains of pain, function, gait, deformities, and range of motion of patients undergoing THA. 

The questionnaire consists of a scale ranging from 0 to 100 points, considering each of its domains. The maximum score for each domain is as follows: pain—44 points; function—47 points (this item is subdivided into Activities of Daily Living (AVD)—14 points (climbing stairs, getting on public transport, sitting down, tying shoes/putting on socks), and gait—33 points (lameness, use of support, and distance)); deformities—4 points; and range of motion (ROM)—5 points. The total score of the HHS is considered bad if the sum is less than 70 points; regular if between 70 and 79 points; good if between 80 and 89; and excellent if between 90 and 100 points [44,45].

### 2.5. Hip Range of Motion Parameters

The goniometer used in this study was the so-called universal goniometer, used manually (Figure 1). We base ourselves on the values followed by the American Academy of Orthopedic Surgeons (1965) and Huang et al. (2020) [46]. All patients were evaluated for hip joint range of motion (ROM) preoperatively and postoperatively (two years).

To measure the range of hip flexion, the older person remained in a supine position, in which the following goniometer positioning was considered: axis to the hip joint, arm fixed; parallel to the longitudinal axis of the trunk, in the line of the greater trochanter of the femur and mobile arm; placed in the lateral midline of the femur (direction of the lateral epicondyle). In the range of hip extension, the same positioning of the goniometer was considered, however, with the older person in ventral decubitus.

To measure abduction and adduction, the axis of the goniometer was positioned on the anterior surface of the hip joint, the fixed arm placed parallel to the anterior superior iliac spine and the mobile arm placed on the anterior surface of the thigh parallel to the anterior midline of the femur, in the midline direction of the patella. As for the measurements of the external and internal rotations, the older adults were positioned in the sitting position and the axis of the goniometer was considered on the anterior surface of the patella, the arm fixed on the anterior line of the tibia, and the arm moving towards an equidistant point between the malleoli.

### 2.6. Statistical Analysis

The sample size was performed considering function outcome, with an effect size of 0.30 (moderate). In total, 65 older patients were needed to provide an 80% power to detect a moderate effect difference between groups, considering a significance level of 0.05. In addition, due to little sample variability in this study, a large sample was not required.

Data normality was tested using the Shapiro–Wilks test. After confirmation, anthropometric, clinical, radiographic, and functional characteristics, before and two years after THA (24 months), were compared using the paired Student t test. For all analyses, a significance level of 5% was considered.

## 3. Results

**Figure 5:**
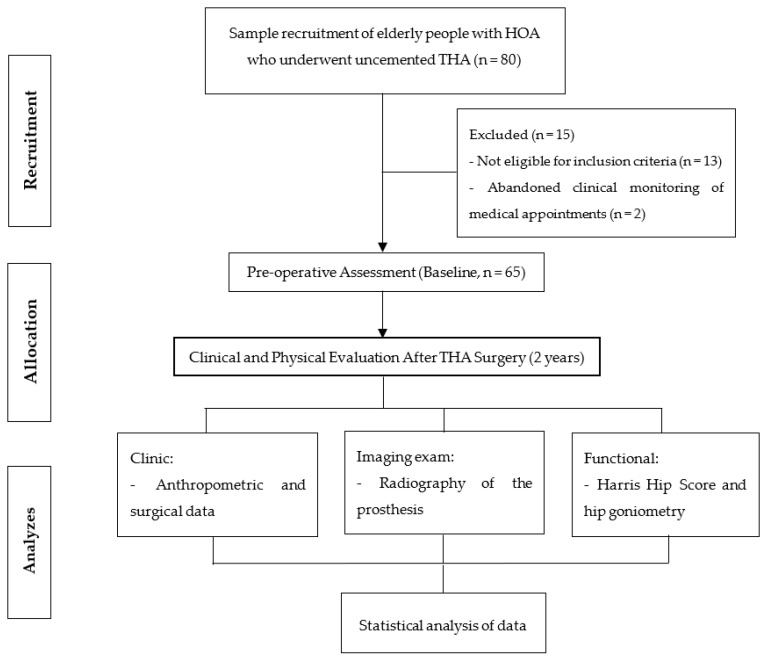
Representation of the protocol flowchart for recruitment and evaluation of elderly people with hip osteoarthritis (HOA) at baseline and after 24 months of THA (two years).

Initially, 80 older patients were recruited, through their medical records, to participate in this study; 15 were excluded, and thus, a total of 65 older patients with osteoarthritis in an advanced stage (chronically dislocated THA, which we define as a dislocation of more than 4 weeks) who underwent cementless THA participated and completed the proposed evaluation process in a 24-month follow-up (Figure 5). 

The groups preoperatively and two years after surgical treatment for THA did not differ in anthropometric characteristics, except for age, given the two-year monitoring time of THA. Another important aspect was the higher prevalence of males compared to females for performing THA in a private hospital, as shown in Table 1.

The older participants did not differentiate in the radiographic parameters before and 24 months after surgical treatment for THA, demonstrating maintenance of the acetabular and femoral positioning angles after prosthesis, as shown in Table 2. These findings reveal the effectiveness of THA surgical treatment using the national acetabular component for older people with severe hip OA.

Regarding the domain aspects of the Harris Hip Score (HHS), it was observed that the pain domain reduced, together with an increase in the functionality and gait of the older adults with THA, as well as the total score of the questionnaire, showing that the national acetabular component proved to be effective for the functionality of older adults in the long-term period of surgery, as shown in Table 3. 

Table 4 shows that the percentage of complications (dislocation, loosening, infection, and inadequate implant positioning) were low two years after THA. Two complications did not result in a reoperation, including one dislocation and one inappropriate positioning. Survivorship free from any revision and post implant complication was 94% at 2 years (95% CI, 88–98).

Regarding hip range of motion, a significant increase can be observed in all movements: flexion, extension, abduction, adduction, and rotation of the older adults 24 months after THA, showing the functional improvement in the hip over time, as demonstrated in Table 5. 

## 4. Discussion

The purpose of this study was to evaluate the clinical, radiological, and functional aspects preoperatively and two years after THA (MD^®^ national acetabular component device, associated with the Phenom^®^ femoral rod) in older patients with hip osteoarthritis. Based on this rationale, the main results showed a higher prevalence of males for performing THA in a private hospital in the south of São Paulo. The older adults evaluated did not present differences in the pre- and postoperative radiographic parameters two years after the surgical treatment of THA, showing maintenance of the acetabular and femoral positioning angles after fitting the prosthesis. Another important finding was the increased functionality and reduced pain in the older adults two years after THA. Hip joint mobility was also significantly increased two years after THA, as well as few complications related to dislocation, loosening, infection, and inadequate implant positioning, in this short period of two years in the monitoring of these patients. These results, of great clinical relevance, as recommended in the literature, with a close evaluation of the components for aseptic loosening, performing revision surgery only on patients with pain and poor function [47].

A population study carried out in the United States showed that the prevalence of symptomatic hip OA remains around 9.2% among adults aged 45 years and over, with 27% presenting radiological signs of the severe disease in women [48]. A systematic review of the prevalence of radiographic OA of the hip demonstrated an increase in the mean prevalence with advancing age for men and women [49]. Men have a higher prevalence of hip OA between 50 and 60 years of age, while women have a higher prevalence after 60 years of age [50]. According to the Centers for Disease Control and Prevention, the lifetime risk for symptomatic and severe hip OA is 18.5% for men and 28.6% for women [15]. In the current study, it was noteworthy that men had a higher prevalence of THA, a fact that can be explained by the studies above, in which men are affected at a younger age than women, favoring a longer time of chronicity of the hip osteoarthritis and, therefore, evolving before surgical treatment with THA, assistance that is of paramount importance in public and private hospitals. 

Another important point observed in this study was the improvement in pre-operative radiographic parameters two years after surgical treatment for THA, showing correct acetabular and femoral positioning after prosthesis, as the implant’s femoral positioning remained adequate in 98% of patients two years after THA. These findings demonstrate the effectiveness of the surgical technique with a cementless prosthesis in older patients with hip osteoarthritis from rheumatologic causes, using national acetabular component material, which is a low-cost system for public and private health hospital care. According to the literature, worldwide, THAs are performed in approximately 1 million patients with hip osteoarthritis each year [51], which is a procedure that demonstrates an effective cost–benefit relationship, especially cemented prosthesis [8,9,10,52], in older adults, whose frailties and functional disabilities can become even more intense with the presence of hip osteoarthritis [4,6]. Although we did not propose to verify the hospital cost in the current study, the material used was national; therefore, it already promotes an effective cost–benefit relationship to the hospital, accounted for by the reduction in expenses from imported materials, which was of great value, as it is a private hospital with great demand for THAs. This importance was also reiterated in a study carried out in England, when observing high demand for assistance and physical-functional health care for patients with hip and knee osteoarthritis with evolution to surgical treatment [7]. 

From the perspective of functional improvement, scientific evidence has focused on the longevity of hip implants to maintain functional capacity and joint mobility for the practice of physical exercises and activities of daily living after performing THA [17,18], since failures in the THA procedure can result in worse clinical-functional outcomes [53]. In the current study, there was an increase in functionality and gait, with a reduction in the symptoms of hip pain in the older adults two years after cementless THA. Furthermore, there was an increase in hip joint mobility, which is of fundamental importance for improving patient ability to perform daily exercise and activity. These findings are in line with a retrospective cohort study, with a 15-year follow-up, including 49 patients who underwent THA with cementless femoral fixation (cobalt-chromium rod with proximal porous coating), in which functionality increased considerably, with a reduction in pain symptoms in the hip and thigh [54]. 

The differential of the present study was to verify the improvement in the patient’s joint mobility and functionality in the short term (two years), especially in older patients with hip osteoarthritis, in addition to having used material from the national acetabular component to perform the cementless THA. In the literature, there is only one study reporting on national material for hip prosthesis, in which 84 young adult patients (47 years old) underwent THA with a cementless Biomec brand prosthesis (manufactured in Rio Grande do Sul, Brazil) and with 10-year monitoring. The results showed increased mobility and functionality and reduced hip joint pain [55]. Although that study evaluated national material with a different brand to the present study and the sample was young adults, we can consider that cementless prosthetization with national material in older adults, in two years, corroborates the findings of Sheidt et al. (2010) [55].

Another important consideration observed in the current study was that, after two years of prosthesis (short term), the complications related to dislocation, loosening, and inadequate positioning of the implant were reduced, without infection rates, which is a topic of extreme importance for the clinical and functional improvement observed in the older patients evaluated. Of the complications observed regarding dislocation, two cases (3%) were verified, both in the immediate postoperative period (less than 1 week after surgery), and both were posterior hip dislocations (the posterior access route favors posterior dislocation). In both cases, the femoral head was 32 in diameter, as the component used only allows a 36 mm head in sizes above 56 mm from the acetabular cup (this size is only used in very large acetabulum). In one of the cases, the dislocation was not due to the acetabular component, but due to a change in the femoral offset, which was revised and the middle head replaced by a long neck, thus solving the patient’s joint instability. In the other case of dislocation, an acetabular malposition was verified (only case of implant malposition—1.5%); the component was revised and repositioned properly. In the only case of loosening verified, the patient had osteolysis around the acetabular component to De Lee zone 3 [43]. Despite the evident radiographic loosening, the patient had low functional demand due to age, few complaints of pain, and, until the conclusion of this study, did not want to undergo component replacement. As all the prostheses used were the ceramic-on-ceramic type, it was not expected that any patient would present wear of the components, since wear of the ceramic is extremely low. Thus, the percentage of complications from THAs in the older patients, after two years (short-time), remained at 6%, showing benefits of the prosthesis and a reduced percentage when compared to a study carried out by Xará-Leite et al. (2021) [56] comparing older adults (mean 72.2 years, with male prevalence) in a 7-year follow-up. The results found were intraoperative complications, such as femoral or acetabular fractures (5.3%), dislocation rates of 2.8%, failure rates of 2% (when there was aseptic loosening of the revised component), and 9.3% implant survival. 

The issue of survival rates of implants after performing THA remains under considerable debate regarding the best method of fixation of the components. A randomized clinical trial, which included 250 patients (mean age, 64 years) with osteoarthritis treated by performing a THA with and without cement, was followed for a median of twenty years (range, seventeen to twenty-one years). The 20-year survival analysis revealed significantly lower survival rates for cemented implants compared to cementless implants. The cementless conical rod had an extremely good survival rate of 99%. The radiographs showed evidence of slight protection against stresses in around 95% of the cemented rods and 88% of the cementless rods; protection against stresses of grade 3 or higher was observed in around 12% of the remaining cementless rods [57]. 

The results obtained in the current study, in the short term, of cementless THA performed in older patients with hip osteoarthritis from rheumatologic causes, using national acetabular component material, are in agreement with those reported in the literature [52,53] and produced good clinical and radiographic results over the two years. However, the limitation of this study was that it did not compare THAs (cemented and cementless), in the etiology of osteoarthritis in affected older people. Future studies comparing cemented and cementless hip surgery in older adults who undergo THA, in the short and medium term, may better help in the clinical-functional understanding of older adults for the practice of exercise, thus avoiding possible persistent sedentary behavior.

## 5. Conclusions

Older patients with hip osteoarthritis, as a rheumatologic cause, who underwent cementless total hip arthroplasty, ceramic in ceramic, using national acetabular component material, which is a low-cost for health hospital care, in a two-year follow-up, showed improving clinical, radiographic, and functional parameters.

## Figures and Tables

**Table 1:** Comparison of anthropometric aspects baseline of the surgical treatment (GPO) and after a two-year follow-up of total hip arthroplasty (THA) in older patients with unilateral osteoarthritis.

Variables	GPO (*n* = 65)	THA (*n* = 65)	*p*
Age (years)	60.3 ± 14.8	62.8 ± 15.0	0.010 *
Mass (Kg/cm^2^)	75.5 ± 10.6	74.5 ± 9.6	0.063
Stature (cm)	1.64 ± 0.5	1.65 ± 0.6	0.164
BMI (Kg/cm^2^)	27.8 ± 4.2	28.1 ± 3.9	0.383
Sex (%)	F (46.2); M (53.8)	F (46.2); M (53.8)	-

* Student’s *t*-test, dependent, significant differences *p* < 0.05.

**Table 2:** Comparison of radiographic aspects baseline of the surgical procedure (GPO) and in the two-year post-operative period of total hip arthroplasty (THA) in older people with unilateral osteoarthritis.

Radiographic Examination	GPO (*n* = 65)	THA (*n* = 65)	*p*
Acetabular placement (degrees)	44.3 ± 5.9	44.4 ± 6.3	0.083
Implant Femoral Offset (cm)	4.5 ± 0.7	4.5 ± 0.6	0.102

**Table 3:** Comparison of functional aspects by the pre-surgical baseline (GPO) and two-year postoperative Harris Hip Score (HHS) domains of total hip arthroplasty (THA) in older people with unilateral osteoarthritis.

HHS Domains	GPO (*n* = 65)	THA (*n* = 65)	*p*
Pain	41.3 ± 3.5	20.7 ± 8.5	0.001 *
Function	9.5 ± 2.1	12.7 ± 1.6	0.012 *
Gait	22.5 ± 5.8	31.1 ± 4.9	0.001 *
Deformity	3.4 ± 1.2	4.0 ± 1.0	0.013 *
Total score	56.3 ± 14.6	85.8 ± 9.6	<0.001 *

* Student *t*-test, dependent, considering statistical differences *p* < 0.05.

**Table 4:** Comparison of functional aspects by the pre-surgical—baseline (GPO) and two-year postoperative Harris Hip Score (HHS) domains of total hip arthroplasty (THA) in older people with unilateral osteoarthritis.

Post Implant Complications	Number (*n* = 65)	Percentage (0–100%)
Inappropriate positioning	1	1.5%
Dislocation (recurrent dislocation)	2	3.0%
Loosening	1	1.5%
Joint infection in two hips (deep periprosthetic)	0	0%
Total	4	6.0%

**Table 5:** Comparison of hip range of motion baseline of the surgical procedure (GPO) and in the late postoperative period after the two-year follow-up of total hip arthroplasty (THA) in older people with unilateral osteoarthritis.

Hip Goniometry	GPO (*n* = 65)	THA (*n* = 65)	*p*
Flexion	36.6 ± 6.1	54.4 ± 11.2	0.001 *
Extension	5.4 ± 1.8	7.7 ± 1.3	0.001 *
Abduction	15.7 ± 3.1	25.9 ± 7.6	<0.001 *
Adduction	6.3 ± 2.4	11.1 ± 1.9	<0.001 *
External rotation	13.3 ± 3.7	25.6 ± 5.3	0.001 *
Internal rotation	11.0 ± 1.6	22.1 ± 5.4	0.001 *

* Student *t*-test, dependent, considering statistical differences *p* < 0.05.

## Data Availability

The datasets generated and/or analyzed during the current study are not publicly available due to limitations of ethical approval involving the patient data and anonymity but are available from the corresponding author (apribeiro@alumni.usp.br) on reasonable request.
